# Rapidly Developing Yeast Microcolonies Differentiate in a Similar Way to Aging Giant Colonies

**DOI:** 10.1155/2013/102485

**Published:** 2013-07-21

**Authors:** Libuše Váchová, Ladislava Hatáková, Michal Čáp, Michaela Pokorná, Zdena Palková

**Affiliations:** ^1^Institute of Microbiology of the ASCR, v.v.i., 142 20 Prague 4, Czech Republic; ^2^Department of Genetics and Microbiology, Faculty of Science, Charles University in Prague, 128 44 Prague 2, Czech Republic

## Abstract

During their development and aging on solid substrates, yeast giant colonies produce ammonia, which acts as a quorum sensing molecule. Ammonia production is connected with alkalization of the surrounding medium and with extensive reprogramming of cell metabolism. In addition, ammonia signaling is important for both horizontal (colony centre versus colony margin) and vertical (upper versus lower cell layers) colony differentiations. The centre of an aging differentiated giant colony is thus composed of two major cell subpopulations, the subpopulation of long-living, metabolically active and stress-resistant cells that form the upper layers of the colony and the subpopulation of stress-sensitive starving cells in the colony interior. Here, we show that microcolonies originating from one cell pass through similar developmental phases as giant colonies. Microcolony differentiation is linked to ammonia signaling, and cells similar to the upper and lower cells of aged giant colonies are formed even in relatively young microcolonies. A comparison of the properties of these cells revealed a number of features that are similar in microcolonies and giant colonies as well as a few that are only typical of chronologically aged giant colonies. These findings show that colony age *per se* is not crucial for colony differentiation.

## 1. Introduction

When developing on solid media or in nonshaken liquid environments, yeast cells can organize into structured and differentiated multicellular communities where individual cells gain specific properties and can fulfill specific roles. Colonies, stalks, biofilms, and flors on liquid surfaces are examples of such organized communities [1–11]. Colonies growing on solid agar medium usually originate either from individual cells (microcolonies) or from a cell suspension spotted onto the agar (giant colonies) [12–14]. The morphology and internal architecture of both microcolonies and giant colonies are dependent on the yeast species or even the strain that forms the colony, the cultivation conditions (e.g., nutrient sources), and developmental phase (i.e., the age of the colony). Thus, for example, natural strains of *Saccharomyces cerevisiae* form structured biofilm colonies [15, 16] that to some extent resemble the colonies formed by pathogenic yeasts of the *Candida* or *Cryptococcus* species [7]. These structured colonies exhibit features (such as the presence of multidrug resistance transporters and an extracellular matrix) that are important for the formation, development, and survival of natural yeast biofilms [17]. The internal architecture of these structured colonies differs strikingly from the architecture of smooth colonies that are formed by laboratory strains of *S. cerevisiae*.

As we have shown previously, giant colonies of *S. cerevisiae* laboratory strains grown on solid complex respiratory medium pass through distinct developmental phases that can be detected by monitoring the pH changes of the medium, changing from the acidic to near alkali and vice versa [13]. The alkali phase of colony development is accompanied by the production of volatile ammonia that functions as a signal important for colony metabolic reprogramming and long-term survival [13, 18–20]. Such metabolic reprogramming appears to be more important for colony survival than some mechanisms eliminating stress factors, such as stress defense enzymes [21]. We have demonstrated that ammonia-related changes are important for diversification between the cells in the center and margin of a colony [20–22]. We have also recently shown that ammonia signaling and related metabolic reprogramming are involved in the diversification of the cells of the colony and the formation of cells with specialized functions precisely localized within the colony [23, 24]. Thus, during the switch of giant colonies to the alkali phase, both horizontal and vertical differentiations occur, where central and margin cells behave differently, as do cells located in the upper and lower regions of the colony center. Detailed analysis of the central colony region revealed two major cell subpopulations located in the upper (U cells) and lower (L cells) colony areas that differ in their morphology, physiology, and gene expression. U cells are large stress-resistant cells with a longevity phenotype, while L cells are smaller, more sensitive to various stresses (such as heat shock and ethanol treatment), and lose viability over the time. Both cell types significantly differ in their gene expression, as shown by a transcriptomic comparison of U and L cells isolated from 15- and 20-day-old colonies [23]. According to these transcriptomic data, U cells seem to be metabolically active cells with induced amino acid metabolism, glycolysis, and some other pathways such as the pentose-phosphate shunt. U cells also express a large group of genes coding for ribosomal and some other proteins of the translational machinery. These genes are usually controlled by the TOR pathway under nutrient-rich conditions. Some other expression characteristics of U cells, however, indicate that some pathways usually active under conditions of nutrient limitation are also induced in U cells and affect their physiology [23]. For example, a large group of amino acid biosynthetic genes is controlled by the transcription factor Gcn4p [25]. In contrast to U cells, L cells behave like stressed cells—they have low metabolic activity and seem to activate some degradative mechanisms that can contribute to the release of compounds that can be exploited by U cells. 

An important question is to what extent chronological aging of the whole colony population on one side and active signaling (which includes the action of ammonia and related metabolic reprogramming as well as other not yet identified signaling and regulatory processes) on the other side contribute to *S. cerevisiae* colony development, differentiation, and long-term survival. As was mentioned above, giant colonies activate ammonia signaling and form U and L cells between days 7 and 10 of colony development when most of the colonial cells are in the stationary (or slow growth) phase. That is, cells differentiating into U and L cells are relatively old and most of them have already persisted in nondividing form for several days. Both of the above-mentioned processes (chronological aging and signal-related metabolic reprogramming) are therefore running in parallel in giant colonies and thus both could contribute to colony differentiation and U and L cell properties. In contrast to giant colonies, switch to the alkali phase and ammonia signaling among microcolonies usually starts much earlier than in giant colonies and central differentiated cells are therefore much younger (i.e., less chronologically aged) in microcolonies than in giant colonies. However, the major expression changes that accompany medium alkalization and ammonia production in microcolonies resemble those changes identified in giant colonies [18]. Similarly, Ato1p, a putative ammonia exporter, is produced in the margin and upper central cell layer in both giant colonies and microcolonies when they begin to alkalize the medium [22, 24]. Here, we examined the main features of the central parts of differentiated microcolonies and compared these features to those described in giant colonies. Through this analysis, we showed that prominent characteristics of central upper cells of yeast colonies are not related to colony aging but dependent on active colony reprogramming. On the other hand, some other features such as in particular the stress resistance of cells in the colony interior (L cells) significantly differ in younger microcolonies compared to giant colonies, being related to colony aging.

## 2. Results and Discussion

### 2.1. Microcolonies Pass through the Same Developmental Phases as Giant Colonies

**Figure 1:**
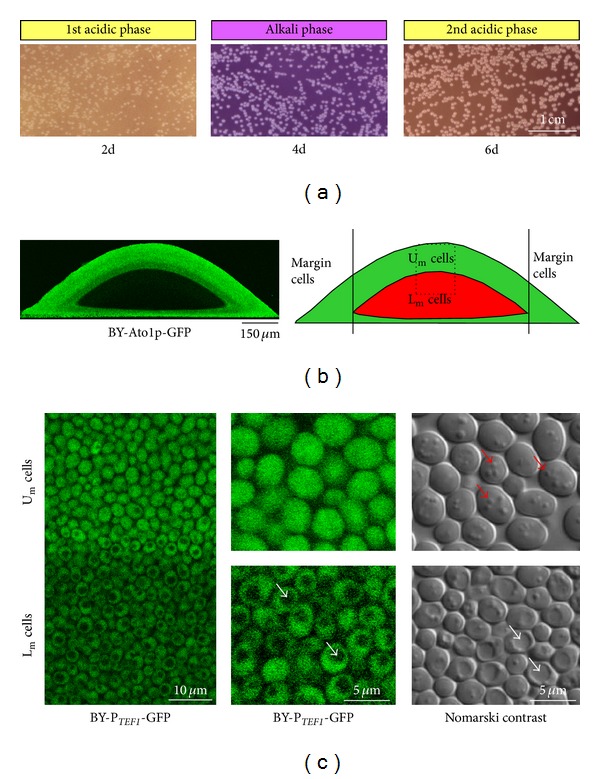
Developmental phases and vertical differentiation of yeast microcolonies. (a) Microcolonies develop on GMA-BKP. BKP functions as pH dye indicator with pKa of 6.3, the color of which changes from yellow at acidic pH to purple in more alkali pH. Microcolonies were in the 1st acidic (2 d), alkali (4 d), and beginning of the 2nd acidic (6 d) phases. Bird views of microcolonies are shown. (b) Vertical transversal cross-section viewed by 2PE-CM of akali-phase microcolony formed by the strain producing Ato1p-GFP (left) and scheme of the localization of three cell subpopulations within the microcolony (right). (c) Boundary between U_m_ and L_m_ cells (left) and morphology of U_m_ and L_m_ cells (center) of BY-P_*TEF*1_-GFP strain at vertical cross-sections of 4-day-old microcolonies analyzed by 2PE-CM. Cytosolic expression of GFP is used for *in situ* visualization of U_m_ and L_m_ cells by 2PE-CM since it enables the visualization of large vacuoles in L_m_ cells (from which the fluorescence is excluded) and the size of U_m_ and L_m_ cells. Morphology of U_m_ and L_m_ cells from 4-day-old BY4742 microcolonies separated by gradient centrifugation and visualized by Nomarski contrast (right). White arrows show large vacuoles in L_m_ cells; red arrows show lipid droplets in U_m_ cells.

Similarly to giant colonies, microcolonies of BY4742 growing on GMA solid plates pass through the developmental phases characterized by changes in external pH and ammonia production [24, 26]. Microcolonies, thus, pass through the first acidic, alkali and second acidic developmental phases (Figure 1(a)), where the alkali phase is accompanied by ammonia production. In contrast to giant colonies, in which the timing of the transition to the ammonia-producing period is typically standardized by the inoculation of six giant colonies on the plate [18], the timing of the acid-to-alkali microcolony transition is dependent on the density of the plated microcolonies. More densely plated microcolonies switch to the ammonia-producing period earlier than microcolonies growing at a lower density on the plate. Like giant colonies that synchronize ammonia production and developmental phases [27], microcolonies on the same plate also synchronize themselves via the ammonia that starts to be produced by the most densely plated microcolonies. For the experiments described in the following sections, we used a standard plating of approximately 5000 microcolonies per plate, that is the density of microcolonies that results in microcolony transition to the alkali phase between days 3 and 4 of colony growth. 

### 2.2. Switch to Ammonia Production Is Accompanied by Differentiation of Microcolonies and Formation of U_m_ and L_m_ Cells

**Figure 2:**
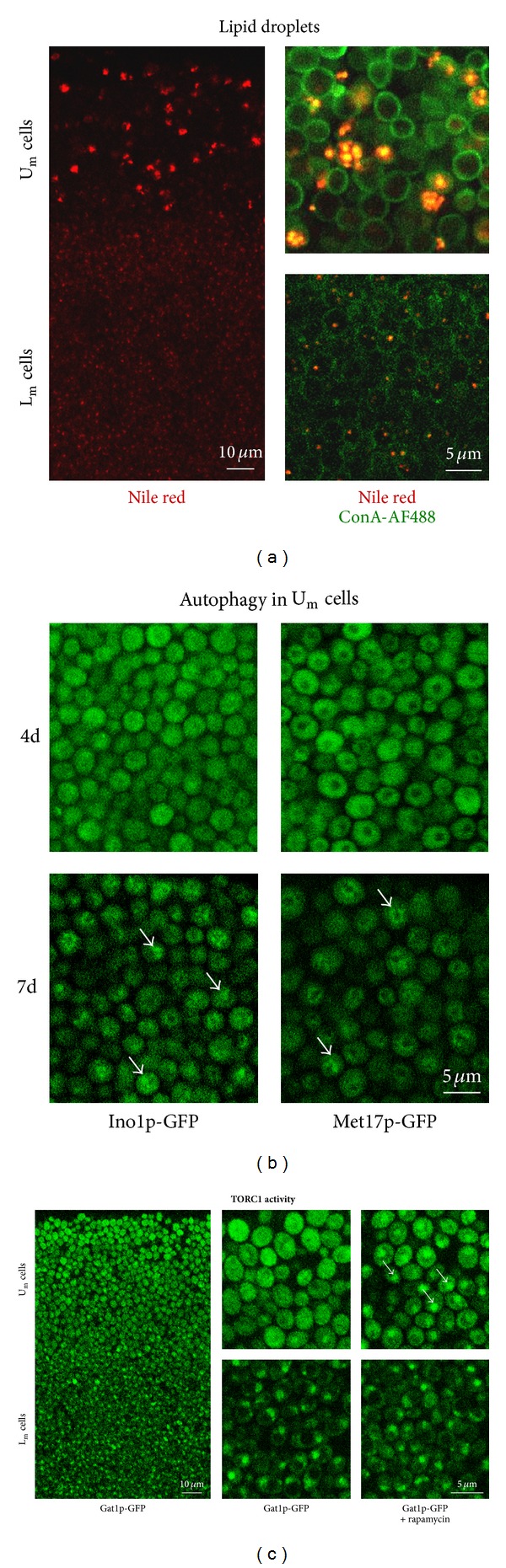
Localization of cells containing storage compounds (lipid droplets) and cells with active autophagy and TORC1 signaling pathway. (a) Vertical transversal cross-sections of 4-day-old BY4742 microcolonies. Left, boundary between U_m_ and L_m_ cells, lipid droplets are stained with Nile red. Right, lipid droplets of U_m_ and L_m_ cells stained with Nile red (red) and cell walls with concanavalin A conjugated with Alexa Fluor 488 (green). (b) Vertical cross-sections of 4- and 7-day-old microcolonies of strains producing cytosolic proteins Ino1p-GFP or Met17p-GFP. U_m_ cells are shown; arrows indicate GFP in vacuoles of 7-day-old U_m_ cells where cytosolic proteins were delivered to vacuoles via autophagy. (c) Vertical cross-sections of 4-day-old microcolonies formed by Gat1p-GFP strain showing localization of Gat1p-GFP protein in U_m_ and L_m_ cells. Arrows indicate relocalization of Gat1p-GFP from the cytosol to the nuclei of U_m_ cells after treating the cut edge of the colony section with 250 ng/mL rapamycin, an inhibitor of TORC1.

As with giant colonies [23], the transition of microcolonies to the alkali phase is accompanied by a diversification of the relatively homogeneous cell population of the 1st acidic phase microcolonies to two major cell types that are localized in the upper and lower layers of alkali phase microcolonies.  Figure 1 shows that these upper and lower cells morphologically resemble the U and L cells of giant colonies, respectively. Cells in the lower parts of a microcolony (L_m_ cells) are smaller and usually contain one large vacuole, while cells in the upper parts (U_m_ cells) are larger with no visible vacuoles. The staining of microcolony sections by Nile red (Figure 2) as well as Nomarski contrast visualization (Figure 1(c)) confirmed that similarly to giant colonies, U_m_ cells contain several large lipid droplets, while L_m_ cells usually contain one small lipid droplet. 

**Figure 3:**
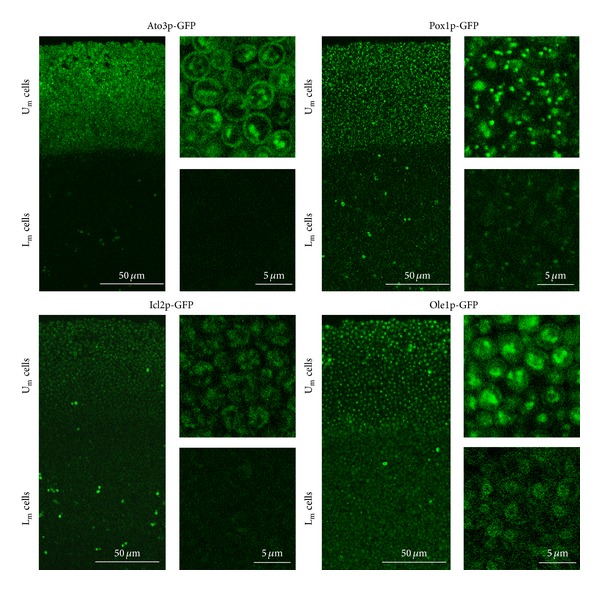
Profile of selected, GFP-labeled proteins in alkali phase microcolonies. 2PE-CM of vertical transversal cross-sections of alkali phase (4-day-old) microcolonies formed by strains producing particular labeled proteins.

In addition to their morphology similarities, we found similar profiles of proteins produced by the U_m_ cells from 4-day-old microcolonies and by the U cells of 15-day-old giant colonies [23]. Hence, all three Ato proteins (Ato1p, Ato2p, and Ato3p) started to be produced exclusively in U_m_ cells but not in L_m_ cells (as shown for Ato3p-GFP in Figure 3) after the microcolonies had entered the alkali phase. Similarly, Pox1p-GFP and Icl2p-GFP are preferentially produced in U_m_ cells (Figure 3) as well as in the U cells of giant colonies [23]. In addition, the production profile of Ole1p-GFP is also similar in microcolonies and giant colonies. Ole1p-GFP is produced and properly localized to the endoplasmatic reticulum (ER) in L and L_m_ cells, respectively, while it is degraded within vacuoles in U and U_m_ cells, respectively, (Figure 3). 

Another typical feature of differentiated giant colonies is an increased activity of TORC1 in U cells and its inactivation in L cells, as shown by the different localization of Gat1p-GFP in the two cell types [23]. The GATA transcription factor Gat1p was shown to be phosphorylated by TORC1, which results in Gat1p cytosolic localization and thus functional inhibition [28]. Confocal microscopy of microcolony cross-sections clearly showed that Gat1p-GFP is localized to the nuclei of L_m_ cells, which indicates that TORC1 is inactive in L_m_ cells (Figure 2(c)). In U_m_ cells, TORC1 is apparently active, as Gat1p-GFP is predominantly in the cytosol (i.e., phosphorylated) and it only moves to the nucleus when a TORC1 inhibitor rapamycin is added to the colony sections.

In summary, these data show that several typical features of the U cells of giant colonies are found in the U_m_ cells of microcolonies that switch to the alkali phase of ammonia production, even though U_m_ cells are far younger chronologically than the U cells of giant colonies. The typical features of U cells, such as the accumulation of lipid droplets, production of typical marker proteins, and active TORC1, are found in U_m_ cells soon after the upper and lower layers have formed in microcolonies entering the alkali phase. These data therefore indicate that ammonia-related signaling events are more significant than chronological age in the formation of these typical features of upper cells. This is in agreement with the previous finding that in giant colonies, the formation of cells morphologically resembling U cells can also be prematurely induced by ammonia from an artificial source [23].

### 2.3. Autophagy Appears Later in U_m_ Cells

Another typical feature of the U cells of giant colonies is active autophagy [23]. Monitoring the cellular localization of GFP in the microcolonies of strains producing cytosolic Ino1p and Met17p labeled with GFP showed a significant vacuolar GFP signal in the U_m_ cells of 7-day-old microcolonies (Figure 2). However, no vacuolar localization of GFP was visible in 4-day-old colonies. As in the L cells of giant colonies, no vacuolar GFP was detected in L_m_ cells of any age. These data showed that U_m_ cells activate autophagy like the U cells of giant colonies. However, the autophagy is initiated later than other typical processes of U_m_ cells and seems, therefore, to be more dependent on the chronological aging of U_m_ cells. 

### 2.4. U_m_ and L_m_ Cells Differ in Their Respiratory Capacity

**Figure 4:**
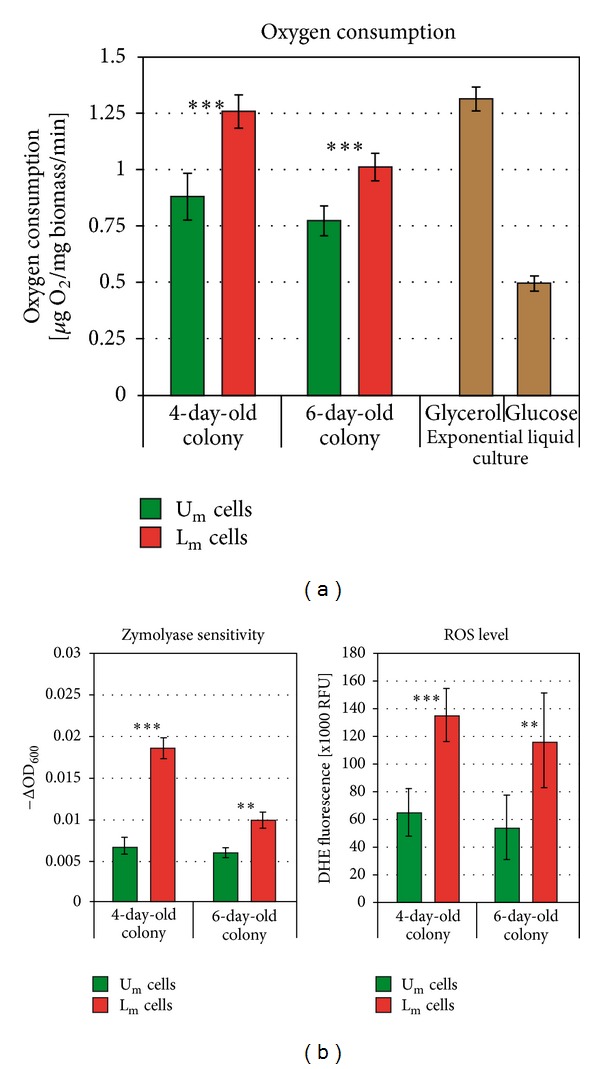
Physiological differences between U_m_ and L_m_ cells from 4- and 6-day-old colonies. (a) Oxygen consumption as a measure of respiratory capacity of U_m_ and L_m_ cells isolated from 4- and 6-day-old microcolonies. Respiration of glucose- and glycerol-grown cells from exponential liquid shaken cultures is shown for comparison. (b) Stress-related features of U_m_ and L_m_. Sensitivity to zymolyase is shown as a decrease in optical density of cell suspensions (left). Production of ROS measured as fluorescence of DHE (right). All data represent averages of at least three experiments ±SD. **—*t*-test *P* value < 0.01; ***—*t*-test *P* value < 0.001.

An important and unexpected difference between the U and L cells of giant colonies is in the capacity of these cells to consume oxygen [23]. Although localized close to the air, U cells exhibit significantly decreased ability to consume oxygen as compared with L cells, and, accordingly, U cells contain large swollen mitochondria with few cristae. On the other hand, L cells maintain their capacity to consume oxygen quite effectively and contain normal-looking cristated mitochondria. To compare the respiration of U_m_ and L_m_ cells, we separated these cells from 4- to 6-day-old microcolonies by gradient centrifugation and measured their respiratory capacity. As shown in Figure 4(a), the U_m_ cells of 4-day-old microcolonies already consume less oxygen than L_m_ cells of the same age. This difference persisted in older colonies. These data show that as with the other features described above, the decreased respiratory capacity of U cells identified in 15-day-old giant colonies and in 4-day-old alkali phase microcolonies is a characteristic that is also most likely predominantly induced by a signaling event and not by the aging of colony. 

### 2.5. L_m_ Cells Differ from L Cells of Giant Colonies in Some of Their Features

Other physiological differences between the U and L cells of giant colonies are in terms of reactive oxygen species (ROS) production, resistance to the cell wall degrading enzyme zymolyase, and sensitivity to various stresses, such as heat shock and ethanol treatment [23]. Measurement of the ROS level in U_m_ and L_m_ cells separated from microcolonies and stained with dihydroethidium (DHE) showed that L_m_ cells produce significantly higher amount of ROS than U_m_ cells. This difference was significant in 4-day-old microcolonies and persisted in older microcolonies (Figure 4(b)). L_m_ cells are also more sensitive to zymolyase treatment than U_m_ cells, thus, indicating a weaker cell wall of L_m_ cells. Thus, the differences in both ROS production and zymolyase resistance between U_m_ and L_m_ cells were similar to those observed between the U and L cells of giant colonies. 

On the other hand, an analysis of U_m_ and L_m_ cells from 4- to 6-day-old microcolonies did not reveal significant differences in the sensitivity of the two cell types to heat shock and ethanol treatment (not shown). In general, both U_m_ and L_m_ cells were slightly more resistant to heat shock than U cells from 15-day-old giant colonies and significantly more resistant than L cells from such colonies (i.e., than cells that exhibit a strong decrease in viability after heat shock and ethanol treatment) [23]. In other words, L_m_ cells from 6-day-old colonies have not yet decreased their resistance to these stresses. 

Another difference between microcolonies and giant colonies was in their levels of certain amino acids in the upper and lower cells. While the level of intracellular glutamine was significantly higher in U than in the L cells of giant colonies, only a negligible difference was observed between the U_m_ and L_m_ cells of 6-day-old microcolonies (and no difference in 4-day-old microcolonies). On the other hand, differences in amino acids such as lysine, alanine, and GABA that are present in higher concentrations in L cells than in the U cells of giant colonies are already detectable in 6-day-old microcolonies. Lysine, alanine, and GABA are present in 2.3, 1.6, and 4.9 times higher concentrations in L_m_ cells than in U_m_ cells, respectively. These values are comparable with L/U ratio of 1.6, 2.2, and 3.6, respectively, for 15-day-old giant colonies [23]. These data indicate that particularly a drop in glutamine in L cells is also connected with the chronological aging or prolonged starvation of colonies.

In summary, the data indicate that L_m_ cells from 4- to 6-day-old microcolonies are in a better physiological condition than the L cells of 15- to 20-day-old giant colonies. The observed decrease in the resistance and viability of the L cells of giant colonies as well as the drop in their glutamine content thus seems to appear later during colony chronological aging and is probably not directly related to the changes induced by ammonia signaling and/or related metabolic reprogramming. This conclusion is also supported by the observation that some of the proteins that started to be produced in the L cells of 15-day-old giant colonies and the production which increases later in 20-day-old giant colonies (such as Ino1p and Met17p) are not yet produced in the L_m_ cells of 4- to 6-day-old microcolonies (not shown). 

## 3. Conclusions

The presented data show that various features typical of the U cells of giant colonies growing on complex respiratory medium and undergoing differentiation during their transition to the ammonia-producing alkali phase (10- to 15-day-old colonies) [23] are also found in U_m_ cells located in the upper layers of alkali phase microcolonies that are only 3 to 4 days old. These features include the production of specific proteins, accumulation of storage material such as lipid droplets, activity of specific regulators such as TORC1, decreased function of mitochondria, low level of ROS, and high resistance to zymolyase, indicating a strengthening of the cell wall. Thus, all of these features of U cells seem to be predominantly related to signaling events and the metabolic reprogramming that accompanies the colony transition from the acidic to the alkali developmental phase [18, 22], rather than to the cell chronological aging. Such signaling processes leading to colony reprogramming could be initiated (by not yet identified mechanism(s)), for example, in the first microcolony that senses a nutrient shortage, that is, in a microcolony that is located in the densest area of plated microcolonies. Ammonia is then the signal that spreads the information about “the need for reprogramming” to the other microcolonies over the whole plate. The ability of ammonia to prematurely induce colonies to ammonia production independently of their current developmental phase [27] guarantees that even the sparsely plated microcolonies become induced and initiate the reprogramming and differentiation while still experiencing nutrient abundance. U_m_ cells gain the major properties of the U cells of giant colonies, although they have spent a much shorter time in the stationary or slow growing phase than U cells.

A comparison of the transcriptomes of “outside” and “inside” cells separated by FACS from 4-day-old microcolonies growing on complex glucose medium [29] showed that some expression characteristics of U cells from giant colonies grown on complex respiratory medium [23] are also found in “outside” cells. These characteristics include the expression of genes coding for ribosomal proteins and proteins of the translational machinery, genes for glycolytic enzymes, genes involved in amino acid metabolism, and some others [29]. Similarly, we observed the production of carbonic anhydrase Nce103p in the upper cell layers of microcolonies growing both on complex glucose [30] and complex glycerol (unpublished data) agar media. These data indicate that the expression of particular genes and activation of specific metabolic pathways could be profitable for cells in the upper layers of yeast colonies.

In contrast to U_m_ cells, only some features of L cells are preserved in L_m_ cells of 4- to 6-day-old microcolonies compared to 15- to 20-day-old giant colonies. These include a higher respiratory capacity, higher production of ROS, higher sensitivity to zymolyase, and the production of some proteins (such as Ole1p). Traven et al. [29] also demonstrated an increased expression of genes required for the activity of the mitochondrial respiratory chain genes in the “inside” cells of 4-day-old microcolonies (cells at a similar position within the colony to L cells) grown on glucose complex medium. All of these features are also typical of the L cells of 15- to 20-day-old giant colonies grown on complex respiratory medium [23]. On the other hand, other features of the L cells of giant colonies are not yet present in the L_m_ cells of 4- to 6-day-old microcolonies. In particular, L_m_ cells do not exhibit an enhanced sensitivity to some stresses such as heat shock and ethanol treatment, which indicates that these cells are in a better physiological condition than much older L cells from giant colonies. These stress-related features seem to be therefore more dependent on the chronological age of L cells and could be also related to the duration of the coexistence of U and L cells. Previous findings suggested that U cells are fed at the expense of L cells [23] which could then lead to a deepening of starvation of L cells over the time and to a consequent decrease in their overall viability in older giant colonies. Similarly, autophagy, which seems to be important for the longevity of U cells [23], is only activated later in U_m_ of 6- to 7-day-old microcolonies. This finding suggests that the regulation of autophagy is partially dependent on signaling events guiding the development of U cells (autophagy is only activated in U cells) but that it is also dependent on the aging and nutrition status of U cells.

## 4. Material and Methods

### 4.1. Strains and Media

*S. cerevisiae* strain BY4742 (MAT*α*, *his3*Δ, *leu2*Δ, *lys2*Δ, *ura3*Δ) was from the EUROSCARF collection. BY4742-derived strains containing proteins (Ato1p, Ato3p, Pox1p, Icl2p, Ole1p, Ino1p, Met17p, and Gat1p) fused with GFP at their C-terminus were constructed as described previously [23, 24]. BY-P_*TEF*1_-GFP strain expressing GFP under the control of constitutive promoter of *TEF1* gene (P_*TEF*1_) was constructed by integration of P_*TEF*1_-GFP-natNT2 cassette amplified from pYM-N21 plasmid [31] into *HIS3* locus of BY4742 strain. Yeast microcolonies were grown at 28°C either on GMA (1% yeast extract, 3% glycerol, 1% ethanol, 2% agar, 10 mM CaCl_2_) or on GMA-BKP (GMA, 0.01% bromocresol purple). For standard experiments, cells were plated at an approximate density of 5 × 10^3^ per plate.

### 4.2. Two-Photon Excitation Confocal Microscopy (2PE-CM)

The microcolony sample preparation and 2PE-CM of transversal vertical cross-section of microcolonies were performed according to [24]. When required, the microcolony cross-sections were stained with Nile red (2.5 *μ*g/mL) and concanavalin A labeled with Alexa Fluor 488 (ConA-AF, 30 *μ*g/mL) as described in [17]. Alternatively, GFP fluorescence was monitored. An SP2 AOBS MP confocal scanner microscope (Leica) fitted with a Ti:Sapphire Chameleon Ultra laser (Coherent Inc.) and 63×/1.20 water immersion plan apochromat objective were used. Excitation wavelength was 920 nm, and emission bandwidths were 470–540 nm for ConA, 580–750 nm for NR, and 480–595 nm for GFP.

### 4.3. Colony Images

Colony images were captured in transmitted light with a Navitar objective and a complementary metal-oxide semiconductor camera (ProgRes CT3; Jenoptik).

### 4.4. Sorbitol Gradient Cell Fractionation

Cells from microcolonies were fractionated into subpopulations by centrifugation as described in [23] with the following modification: instead of sucrose, a 10–35% sorbitol gradient was used to avoid changes that could be induced by sucrose in the relatively young cells of microcolonies.

### 4.5. U and L Cell Resistance to Stresses

Cell resistance was assayed using 10-fold serial dilutions of cell suspensions (OD_600_ = 10) that were incubated at 52°C for 45 or 90 min or in 20% ethanol for 60 min and compared to untreated controls. Zymolyase resistance was determined as the decrease in the OD_600_ of a cell suspension (starting OD_600_ = 0.5) in 50 mM potassium phosphate buffer, pH 7.5 with 2 mM dithiothreitol, and 5 U/mL zymolyase (MP Biomedicals).

### 4.6. Respiration Rate and ROS Quantification

The oxygen consumption of 5 mg of freshly isolated U_m_ or L_m_ wet cell biomass was determined at 30°C in 1 mL of water using a 782 oxygen meter with a 1-mL MT-200A cell (Strathkelvin Instruments). ROS was quantified using DHE staining according to Čáp et al. [21] with minor modifications. Briefly, isolated U_m_ and L_m_ cells were resuspended in water to a final concentration of 100 mg/mL. 7.5 *μ*L of this suspension was incubated with 42.5 *μ*L of water and 5 *μ*L of 25 *μ*g/mL DHE solution (freshly prepared from 1 mg/mL stock solution in DMSO). Cells were stained for 25 min in the dark and diluted with 1.95 mL of water, and the DHE fluorescence was measured using a FluoroMax 3 spectrofluorometer (Jobin Yvon) with excitation/emission wavelengths of 480/604 nm. 

### 4.7. Amino Acid Concentration

Total intracellular amino acids were extracted from cell suspensions in water by boiling for 5 min, and the concentration was determined by HPLC with precolumn derivatization by OPA [23, 32] with a ZORBAX Eclipse AAA, 3.5 mm, 4.6 × 75 mm reverse phase column (Agilent), and fluorescence detection.
